# Duloxetine: The Next Gold Standard for Treating Depression and Motor Symptoms in Parkinson’s Disease?

**DOI:** 10.62641/aep.v53i3.1864

**Published:** 2025-05-05

**Authors:** Guilherme Nobre Nogueira

**Affiliations:** ^1^Internal Medicine Department, UFC - Universidade Federal Do Ceará, 60455-760 Fortaleza, Ceará, Brazil

Dear Editor,

I read with great interest the article by Wang *et al*. (2024) [1] on the retrospective analysis of duloxetine’s effectiveness and safety in treating comorbid depression in Parkinson’s disease (PD). The study sheds light on an important aspect of PD management, addressing both motor and non-motor symptoms, especially depression—a common and impactful comorbidity in this population. However, several key points from the study stand out.

Firstly, the superior efficacy of duloxetine, as evidenced by improvements in the Unified Parkinson’s Disease Rating Scale (UPDRS), Hamilton Depression Rating Scale (HAM-D), and Beck Depression Inventory (BDI), is particularly noteworthy. These findings align with previous research suggesting that serotonin and norepinephrine reuptake inhibitors (SNRIs), like duloxetine, offer broad-spectrum benefits by addressing both mood disturbances and motor symptoms. The improvements in motor function, including better gait speed and reduced tremors and rigidity, are a remarkable advantage, especially since many antidepressants have been associated with worsening motor outcomes in PD patients​ [2].

Secondly, the study’s focus on cognitive function is also of great relevance. PD is associated with cognitive decline, and the observation that duloxetine improved scores on cognitive tests such as the Symbol Digit Modalities Test and the Montreal Cognitive Assessment adds significant value to this treatment option. This highlights duloxetine’s potential to provide a more holistic approach to managing PD [3].

Thirdly, one area that could benefit from further exploration is the safety profile of duloxetine. Although the study found no significant difference in the incidence of adverse events compared to traditional treatments, the higher, albeit not statistically significant, rates of nausea, insomnia, and fatigue in the duloxetine group warrant attention. The balance between efficacy and tolerability is particularly important in PD management, where polypharmacy and drug interactions are common concerns​ [2, 3].

In conclusion, Wang *et al*. [1] provide compelling evidence supporting the use of duloxetine in PD patients with comorbid depression. The drug’s dual impact on mood and motor function represents a significant step forward in comprehensive PD care. I encourage future studies to further investigate the long-term safety of duloxetine, particularly in patients with advanced PD and those on multiple medications, to ensure its broader clinical applicability.

## Availability of Data and Materials

Not applicable.
